# The Treatment of Some Cases of Cancer of the Skin

**Published:** 1899-06

**Authors:** B. M. Hutchins

**Affiliations:** Atlanta, Ga.


### THE TREATMENT OF SOME CASES OF CANCER OF THE SKIN.*

By B. M. HUTCHINS, M.D.,

ATLANTA, GA.

I have selected from my records a few cases illustrative of the different methods employed in the treatment of cancer of the skin. I do not wish to extol or prefer any special method of treatment, but simply give my experience with the different kinds and show that success depends solely upon the removal or destruction of all diseased tissue.

One case was that of a robust maiden lady, aged 40. A nickel, five-cent-piece-sized, typical epithelioma occupied the left half of the upper lip from the carmine border and oral angle to half way to the left ala nasi. A part was ulcerated, the rest firm, waxy.

Marsden's (arsenic) paste was left on for twenty-four hours. It caused much swelling of the lip and left cheek, and changed the diseased parts to a brown-black color. The patient returned to her home in South Carolina and the lesion apparently healed in a month. One year and ten months later the patient returned for treatment.

*Read by title at meeting of State Medical Society at Macon, April, 1899.

Old site of lesion all diseased, ulcerated, the growth being now perceptible on the mucous side of the lip, the whole thickness of the lip being involved. The lesion had extended a bit beyond and below the left oral angle. Under chloroform anesthesia the left half of the lip and the left oral angle were excised. The edges of the wound were brought together with four silver wire sutures and then four superficial sutures of silk. Healing was uneventful and a very faint scar was left. I heard directly from this patient thirteen months later. She was entirely well. Three or four years later I heard indirectly that she remained well.

The following case was that of a dentist, aged 51. Partly unbroken and partly ulcerated epithelioma occupying the root of left zygoma, the tragus, and auricle opposite to the helix. A non-ulcerating epithelioma about the base of the right tragus. The lesion on the left side was removed (ether anesthesia) with knife, scissors, and curette according to the usual method. The one on the right was destroyed with the Paquelin cautery. The treatment of the right lesion was thorough and proper; that of the left was not so thorough, and later the cautery and caustic potash were both used. It is doubtful if the lesion on the left was amenable to thorough treatment, owing to its situation.

Four or five years later I heard that the patient was alive, but I did not learn anything of his condition.

Another case, recognized as hopeless before treatment was undertaken, was that of a lady, aged 70. It was a characteristic infiltrated and ulcerating cancer of the right side of the bridge of the nose and cheek adjacent. In this case the knife was not to be considered. Cocain anesthesia was employed and an effort made to destroy the disease with caustic potash. This failed and the patient was literally put to sleep with a half-grain hypodermic of morphine and then Marsden's paste kept on for twelve hours. When the slough separated the disease was found to be much deeper than anticipated, the bone being destroyed. This was one of the cases of which it could be said there was no hope without treatment and little with treatment.

I have used the Paquelin cautery quite frequently for the destruction of the more limited cancers of the skin. Its action is very satisfactory, but the instrument itself is so unreliable that I have lately preferred some other method.

For not too extensive lesions I have used the caustic potash stick because it is effective, convenient, and the pain is transitory. This method has been used in the large majority of my cases, many of which have remained well up to this time, from one to four years, and in fact a return of the disease has been of the greatest rarity.

Of course many of these cases could have been cured by excision, with no scar, but an excision is often impracticable.

The case of a man 40 years old. From the center of the lower lip to near the left oral angle there was a characteristic epithelioma. It was not possible to do a satisfactory V operation as too much tissue had to be removed. There was no evidence of glandular involvement. As the patient had only a few hours in the city it was decided to do the operation under cocain. A 2 per cent. solution was injected along the line of proposed incision, first half way between the skin and mucous membrane, then subcutaneously. The operation was like a circumcision, a curved excision of the growth and a margin of apparently healthy tissue one half inch wide. The skin and mucous membrane were stitched together. Although the incision extended to the frenum of the lower lip the patient could almost close the mouth immediately after the suturing. A collodion dressing was the only practicable one. I have no information regarding the subsequent history of this case.

A pin-head sized epithelioma on the left side of the end of a physician's nose seemed favorable for treatment by electrolysis. Three-fourths to one milliampere was used and the lesion given a thorough negative electrolysis. This failed and the lesion was finally permanently destroyed with caustic potash.

It is a curious fact that the majority of my cases of epithelioma of the cartilaginous part of the nose have shown the growth on the left side, near the tip.

The case of a gentleman of 70 years illustrated multiple, widely separated epitheliomata. In May, 1896, another physician removed about half the right ear for the disease. There has been no return in this part. Early in 1898 I destroyed, apparently, a small lesion on the left side near the end of the nose, using caustic potash. Within six months it was necessary to reapply the caustic. The nose has now been well nine months, but there is a suspicious lesion over the left malar bone.

Case of a robust farmer aged 71. The disease originated in a mole. On the right side of the back of the neck, over the middle of the anterior border of the trapezius was an epithelioma the diameter of a quarter dollar, movable with the skin and showing no glandular involvement. An eighth of an inch below was an atheroma the size of a finger tip. Under chloroform anesthesia and thorough antisepsis, the two lesions were excised, with the skin and fascia, to the surface of the muscle. The incisions were longitudinal to the neck and an inch clear of each border of the cancer. The wound was an ellipse three inches wide and six inches long, and was sutured from end to end.

This operation was done in August, 1897, and the patient remained well when last heard from, a year and a half later.

The case of a lady, aged 36, was interesting. The disease lay in the angle between the nose and left lower orbital border, being a finger-end sized cavity. This patient had been insane as the result of treatment by pastes and plasters. Knowing the impossibility of radical treatment I undertook to keep the growth in check by antisepsis and cleanliness. The success of this management was shown by there being no extension of the lesion in the next four or five months, while the case was under observation. I also tried various lauded new or old "cures," such as formalin, absolute, to 30 per cent. alcoholic injections and the local application of calcium carbid. None of these did any positive good, some did harm. The antiseptic treatment did all that was done to benefit the condition.

Cancer, as usually understood, is an open "eating," destructive sore. The condition present is one of carcinoma plus infection with pus organisms. Destruction of and keeping out pus germs accomplishes much in prolonging the life of the patient and rendering his existence less miserable and offensive.

It may be of interest to give, briefly, the details of the different methods of treatment employed. In cases where excision is practiced, the direction of the wound should be parallel to the normal lines or folds of the skin. The distance which the incisions shall be beneath and beyond the visible borders of the growth must be left to the judgment of the operator, guided by pathological principles. A wide margin of apparently healthy tissue must be removed and a deep dissection made beneath the lesion in all cases. In the dissection, care should be had not to cut into the diseased growth lest implantation of cancer cells into healthy tissues follow. If adjacent glands are involved they must be removed *en masse* with the primary growth.

The thermocautery is used at a cherry-red heat, all visible disease being destroyed, in doing which a capsule of apparently healthy tissue becomes secondarily necrosed. This is a troublesome, and to a watchful patient, barbarous-looking treatment. Healing is slow, but if the wound is properly managed the scar is soft and not like that of an ordinary burn.

Marsden's arsenic-paste I have rarely used, because it is a most tediously acting and continuously painful treatment. The advantages claimed for it have not been fully demonstrated to my mind, and I have seen no statistics showing it superior to caustic potash or even the thermocautery.

The paste is made of equal, or slightly varying, proportions of Arsenious Acid and pulverzied gum acacia mixed with water. Many of those who use it cut or curette away any redundant cancerous tissue and then apply the paste on cloth, extending a little over the visible margins of the lesion. Cocain, locally used, affords but little relief from the pain. The paste is left on for from twelve to twenty-four hours, as required. The eschar and wound are treated on general principles.

The stick of Caustic Potash is my preference among the caustics, and for several years I have rarely used any other. If the cancerous mass is quite thick a little time will be saved and pain spared by the instrumental removal of part of the growth. A two to four per cent. solution of cocain may be injected to moderate the pain. The surrounding tissue is protected by the application of acetic acid, and the same drug may be used to neutralize the action of the potash when it has gone far enough. The lesion is thoroughly destroyed by boring in the stick of caustic potash, or, in very simple lesions, the application of a few drops of the deliquescing chemical. We change to a gelatinous substance all apparent disease, and secondarily necrose a capsule of tissue, which should contain all the outlying cells. The eschar separates in from one to two weeks, and healing then proceeds as in any other wound. However deep this may be, it fills rapidly, under proper management, and the resulting scar is remarkably superficial and pliant.

Since I have used this chemical in the corner of the eye without damage to the ball, and on the gum without injury to adjacent parts, I feel that there is no visible place where it may not safely be used, if properly handled.

*305 and 306 Fitten Building.*

---

### POSTHUMOUS DIPLOPIA.

There was a young doctor of Cork,
Who used to eat corn-meal and pork,
But now he cuts muscles,
And hurries and hustles,
And eats pie with a gold-plated fork.

He looks quite euphoric and wise,
Makes big mon. by the Traffic he plies;
He cures epilepsy,
The piles and dyspepsy,
By carpenter work—on the eyes.

But when all is over and done,
The devil will have his own fun!
He'll make him see double—
Two cauldrons a-bubble—
Two hells where there is but one!

—*P. O. Redston in Philadelphia Medical Journal.*

